# Early Pregnancy Exposure to Ambient Air Pollution among Late-Onset Preeclamptic Cases Is Associated with Placental DNA Hypomethylation of Specific Genes and Slower Placental Maturation

**DOI:** 10.3390/toxics9120338

**Published:** 2021-12-06

**Authors:** Karin Engström, Yumjirmaa Mandakh, Lana Garmire, Zahra Masoumi, Christina Isaxon, Ebba Malmqvist, Lena Erlandsson, Stefan R. Hansson

**Affiliations:** 1 EPI@LUND, Division of Occupational and Environmental Medicine, Department of Laboratory Medicine, Lund University, SE-22362 Lund, Sweden; 2 Environment Society and Health, Division of Occupational and Environmental Medicine, Department of Laboratory Medicine, Lund University, SE-22362 Lund, Sweden; Yumjirmaa.Mandakh@med.lu.se (Y.M.); Ebba.Malmqvist@med.lu.se (E.M.); 3 Department of Computational Medicine and Bioinformatics, University of Michigan, Ann Arbor, MI 48104, USA; lgarmire@umich.edu; 4 Division of Obstetrics and Gynecology, Department of Clinical Sciences Lund, Lund University, SE-22185 Lund, Sweden; Zahra.Masoumi@med.lu.se (Z.M.); Lena.Erlandsson@med.lu.se (L.E.); Stefan.Hansson@med.lu.se (S.R.H.); 5 Ergonomics and Aerosol Technology, Department of Design Sciences, Lund University, SE-22362 Lund, Sweden; Christina.Isaxon@design.lth.se; 6 Obstetrics and Gynecology, Skåne University Hospital, SE-22184 Lund, Sweden

**Keywords:** ambient air pollution, preeclampsia, global DNA methylation, RNA sequencing, epigenetic age, placenta

## Abstract

Exposure to ambient air pollution during pregnancy has been associated with an increased risk of preeclampsia (PE). Some suggested mechanisms behind this association are changes in placental DNA methylation and gene expression. The objective of this study was to identify how early pregnancy exposure to ambient nitrogen oxides (NO_x_) among PE cases and normotensive controls influence DNA methylation (EPIC array) and gene expression (RNA-seq). The study included placentas from 111 women (29 PE cases/82 controls) in Scania, Sweden. First-trimester NO_x_ exposure was assessed at the participants’ residence using a dispersion model and categorized via median split into high or low NO_x_. Placental gestational epigenetic age was derived from the DNA methylation data. We identified six differentially methylated positions (DMPs, *q* < 0.05) comparing controls with low NO_x_ vs. cases with high NO_x_ and 14 DMPs comparing cases and controls with high NO_x_. Placentas with female fetuses showed more DMPs (N = 309) than male-derived placentas (N = 1). Placentas from PE cases with high NO_x_ demonstrated gestational age deceleration compared to controls with low NO_x_ (*p* = 0.034). No differentially expressed genes (DEGs, *q* < 0.05) were found. In conclusion, early pregnancy exposure to NO_x_ affected placental DNA methylation in PE, resulting in placental immaturity and showing sexual dimorphism.

## 1. Introduction 

Preeclampsia (PE) affects, in average, 0.2%–9% of pregnancies worldwide [1,2] and has been associated with increased risk of maternal mortality [3,4] and maternal cardiovascular disease (CVD) in later life. In addition, offspring born to PE mothers are at high risk of low birth weight at birth and CVD later in life [5,6]. Preeclampsia presents with new onset hypertension after 20 weeks of gestation accompanied by proteinuria and/or dysfunction of one or more maternal organs [7]. The pathogenesis of PE is not fully understood, but placental dysfunction due to syncytiotrophoblast stress explains the underlying mechanism of the clinical presentation of PE [8]. Preeclampsia is commonly divided into early-onset PE (manifesting before week 34) and late-onset PE (at or after 34 weeks) [9]. Early-onset PE is characterized by poor placentation and higher maternal and fetal morbidity and mortality. Late-onset PE makes up 80% of the PE cases and is associated with maternal risk factors such as metabolic syndrome, obesity, and impaired glucose tolerance. Studies have implicated that a female fetus is more common in early-onset PE [10] and a male fetus in late-onset PE [11].

PE is a “multifactorial syndrome” in which ambient air pollution may play a role [12,13,14,15,16]. Ambient particulate air pollution is the fourth leading risk factor for attributable deaths and the seventh leading risk factor for attributable burden of disease [17]. Systematic reviews and meta-analyses suggest that exposure to ambient air pollution during pregnancy is associated with increased risk of PE [13,18], especially for the first trimester [18]. However, the underlying mechanism of the association between ambient air pollution during pregnancy and increased risk of PE is not clearly elucidated.

Placental DNA methylation may be one of the underlying mechanisms linking PE to ambient air pollution. DNA methylation occurs predominantly on cytosines (C) that are followed by guanine (G) residues, referred to as CpG sites, that, along with other epigenetic modifications, regulate gene expression [19]. DNA methylation plays a critical role in cell differentiation and epigenetic reprogramming during embryonic development in the first trimester [20]. Environmental exposures occurring at this critical period have a potential to induce additional epigenetic changes and alter gene expression, resulting in increased susceptibility to disease [21,22]. In previous studies, altered DNA methylation in PE placentas has been seen, which was presumed to be associated with placental development and function [23]. In addition, a differential DNA methylation placental profile has been found in relation to exposure to ambient air pollution during pregnancy [24].

Nitrogen oxide (NO_x_) is a generic term for gaseous oxides containing nitrogen including nitric oxide (NO) and nitrogen dioxide (NO_2_) gas. The main source of these gases is internal combustion engines that burn fuels such as oil, diesel, gas, and organic matter at high temperatures. The health effects of NO_2_ have recently been reviewed by WHO with strong evidence of mortality effects at levels above 10 µg/m^3^ [25]. Prenatal exposure to NO_x_ during the first and second trimesters has been associated with hypomethylation of two CpGs located at the Adenosine receptor A2b (*ADORA2B*) gene, suggested to be involved in PE pathogenesis [26]. However, no studies have yet evaluated the association between exposure to NO_x_ during pregnancy and DNA methylation among PE cases and normotensive pregnancies.

The placenta grows rapidly throughout gestation and matures within the limited time in a physiologic phenomenon known as placental ageing [27]. Timely ageing of the placenta is needed for optimal fetal growth and development. The biological age of a tissue can be estimated with high accuracy using epigenetic data from whole-genome DNA methylation arrays [28], and there are specific epigenetic clocks for the placenta that calculates gestational aging [29].

The present study aimed to identify how early pregnancy exposure to ambient nitrogen oxides (NO_x_) among PE cases and normotensive controls influence DNA methylation and gene expression.

## 2. Materials and Methods

### 2.1. Study Population 

**Figure 1:**
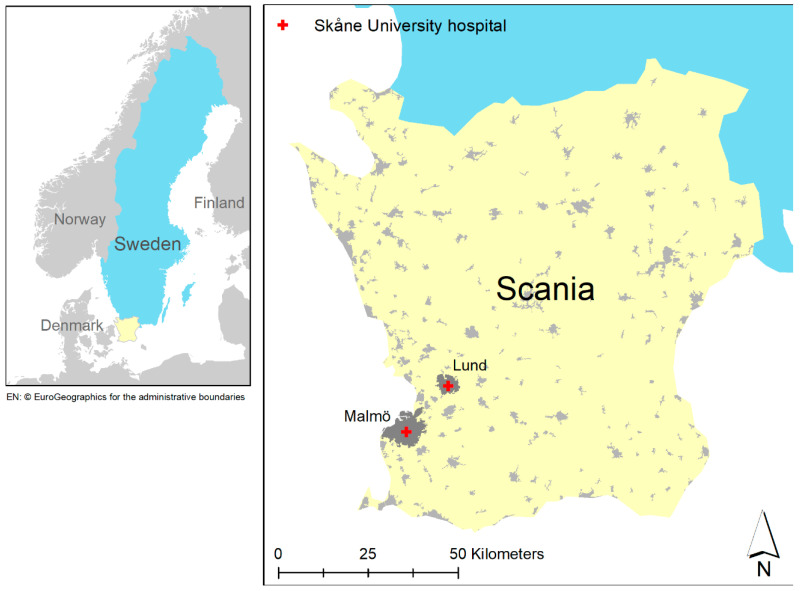
Study setting in Scania, Sweden, showing the two recruitment hospitals.

This study included placentas from pregnant women enrolled in a biobank specifically created for the research on PE at the Department of Obstetrics and Gynecology, Lund University, Sweden. The biobank consists of a biomedical database with biological research samples and detailed clinical data linked to Swedish personal identification numbers for each participant. The placental biopsies used in this study were collected from both Caesarean and vaginal deliveries in 2008–2015 at Skåne University Hospital in Lund and Malmö, Sweden (Figure 1). After excluding those who had multiple pregnancies, smoked during pregnancy, and lived outside the territory of our dispersion model, a total of 111 women were included in the study. Of these, 29 were preeclamptic (one was early-onset and 28 late-onset) and 82 were normotensive. Complete data on NO_x_ exposure during the first trimester, PE status, and potential confounding variables are presented in Table 1. In the descriptive statistics (Table 1), the χ2 test for categorical variables and t-test for continuous variables were used to assess the differences between PE cases and normotensive controls. A p-value of less than 0.05 was considered statistically significant. The study protocol was approved by Lund University Ethical Review Board, LU803-2 (2015/14), Lund, Sweden. Written informed consent was obtained from all participants.

### 2.2. Air Pollution Exposure Assessment 

After selecting study participants with available placental samples in the biobank, we obtained the geocoded residential addresses in Scania, Sweden from the regional population registry using the personal identification numbers. Hourly concentrations of NO_x_ during pregnancy were modeled at geocoded residence level using a modified Gaussian-plume dispersion model with local emission data in EnviMan software [30]. We were not able to model NO and NO_2_ separately, because only NO_x_ emission data was available at the time of exposure assessment. Detailed exposure assessment using the dispersion model has been described previously [31]. The model provides hourly concentrations on 100 × 100 m grids. To account for long-range transboundary air pollution, a background concentration of 2.5 µg/m^3^ was added to the modeled NO_x_ concentrations. The mean exposure to NO_x_ during the first trimester for each person was calculated as the average of the modeled hourly NO_x_ concentrations. Mean NO_x_ exposure was then used as a dichotomized variable via the median cut-off (12.1 μg/m^3^; Interquartile Range: 7.9, 19.1) in high and low exposure groups.

### 2.3. Study Design 

The study participants were divided into groups based on a combination of pregnancy status (PE or normotensive control) and exposure to ambient NO_x_ (low or high NO_x_ exposure based on median split). The study participants were thus divided into four categories: controls with low NO_x_ exposure (N = 45), controls with high NO_x_ exposure (N = 37), PE cases with low NO_x_ exposure (N = 10), and PE cases with high NO_x_ exposure (N = 19). DNA methylation was compared between these groups.

In the gene expression analyses, we only compared PE cases to controls, since the number of individuals (N = 17) included were too low for more in-depth evaluation of differential gene expression.

### 2.4. Association between NO_x_ Exposure and PE 

Associations between NO_x_ exposure during the first trimester (low or high exposure) and PE status was estimated with crude and adjusted logistic regression. The final model was adjusted for clinical data, such as maternal age, pregestational body mass index (BMI), gestational age (linear), and fetal sex [31,32,33,34].

### 2.5. Biological Sample Collection 

The placentas were collected within 4 hours after delivery and stored at 4 °C until biopsies were taken. Briefly, a placental biopsy of 1 × 1 × 1 cm size was excised 7 cm from the umbilical cord insertion or from a central part of the villi region if cord insertion was asymmetrical. Vertically, the biopsy was taken centrally at the thickest point to not include chorionic plate or maternal tissue. Due to the heterogeneity of placental tissue, we also adjusted for the estimated proportion of placental cell types in the statistical analyses (described in Section 2.10). After rinsing in phosphate-buffered saline solution, the placental biopsies were patted dry with a napkin and stored on dry ice for 30 min prior to transfer to −80 °C until further analysis.

### 2.6. DNA and RNA Extraction 

Total DNA and RNA from placental biopsies were extracted using Qiagen AllPrep DNA/RNA/Protein Mini Kit (Qiagen, Hilden, Germany). The DNA and RNA quality (A260/280 and A260/230) were evaluated using a NanoDrop Spectrophotometer ND-1000 (NanoDrop technologies, Wilmington, DE, USA). The RNA integrity was evaluated with LabChip (LabChip GX/GXII systems, PerkinElmer, MA, USA) resulting in 17 samples with an RNA integrity number (RIN) above 7. Only samples with RIN >7 were included in the RNA-seq analyses.

### 2.7. Placental DNA Methylation Laboratory Analyses

A total amount of 500 ng DNA was bisulfite-treated using the EZ DNA Methylation kit v 1.1 (Zymo Research, Irvine, MA, USA). DNA samples were randomized for distribution in two 96-well analysis plates prior to analysis. Genome-wide DNA methylation was determined at the Center for Translational Genomics (CTG), Lund University, Sweden, using the Infinium MethylationEPIC BeadChip (lllumina, San Diego, CA, USA), analyzing approximately 850,000 specific markers of DNA methylation. All beadchips were from the same batch.

### 2.8. Placental DNA Methylation Preprocessing

The statistical software R, version 4.0.208 (R Project for Statistical Computing), was used for thte preprocessing and analysis of the data. The R packages ChAMP [35,36] and minfi [37,38] were used for image processing, quality control, filtering, and normalization. Normalization was performed using the beta-mixture quantile (BMIQ) normalization [35,39]. Non-CpG probes and multi-hit probes were filtered. The detection p-value was below 0.01 for at least >98% of CpGs for all samples. The CpGs with detection *p*-value above 0.01 in more than 20% of the samples were removed.

Analyses of differentially methylated positions (DMPs) were first performed including all samples irrespective of fetal sex. Group comparisons that had significant DMPs were then analysed and stratified for fetal sex. For the analysis in which all individual samples were included, probes in the sex chromosomes were removed. Overall, 805185 CpGs were retained in the analyses including all individual samples, and 823536 CpGs were analyzed in the sex-stratified analyses.

The placental DNA methylation at specific CpG sites was used to estimate the proportion of different placental cell types (Trophoblasts, Stromal, Hofbauer, Endothelial, nucleated RBC, Syncytiotrophoblast) using the R package Planet [40]. Here, reference data from the first trimester were used to infer cell composition using the constrained projection approach implemented in minfi. These estimated cell type proportions were later evaluated as potential covariates in the statistical models.

### 2.9. Predicted Gestational Age Acceleration/Deceleration and Associations with PE and NO_x_ Exposure

The predicted gestational age (GA) was estimated using GA clocks for placental DNA methylation data [29] using the package Planet [40]. By regressing the predicted GA to the chronological GA, the residual gives each placenta a value for GA acceleration/deceleration [41]. A positive value indicates that the placenta ages faster than expected, i.e., an accelerating placental epigenetic clock or GA acceleration. A negative value indicates that the placenta ages slower than expected, which is GA deceleration. The so-called “control placental clock” (CPC) was used [40].

The correlation between predicted GA and chronological GA was evaluated using Spearman correlation for all individuals as well as stratified for PE status. The differences in GA acceleration/deceleration between PE cases and controls, as well as combined PE status and NO_x_ exposure groups (categorical variable), were analysed using linear regression.

### 2.10. DNA Methylation Preprocessing and Data Analysis

Singular value decomposition (SVD) was performed in ChAMP to identify the technical and biological variables associated with DNA methylation (data not shown). These variables were then included in ComBat adjustments [42,43] using ChAMP (for technical variables) or considered as covariates in linear models. The SVD analyses showed that the technical variables “sentrix position” and “analysis plate” accounted for a substantial fraction of variation (*p* < 0.05) in principal components (PCs) one and two, respectively. ComBat adjustments were performed to remove the technical variation. The SVD plots also showed that all estimated cell type proportions had a *p* < 0.05 in the first and/or second PC.

We used estimated fractions of syncytiotrophoblast and Hofbauer cells in the final model, considering that these two cell types form a majority of the placental cell population. Villous syncytiotrophoblasts make up the multinucleated syncytia and are the major cell type in the placenta [44]. Hofbauer cells are located in the placental villous core and are suggested to be involved in immune tolerance during pregnancy [45].

Pregestational BMI, gestational age, fetal sex, and DNA concentration also showed a *p* < 0.05 in the first or second PCs and were thus included in the model. For the sex-stratified analyses, we performed a model with fewer covariates due to a lower number of individual samples included. Sex-specific models were adjusted for estimated fractions of syncytiotrophoblast, Hofbauer cells, DNA concentration, and gestational age.

The DMPs were evaluated by fitting a linear regression model to each CpG using the R package limma [46], with adjustments as described above. M-values (the log_2_ ratio of the intensities of methylated probe versus unmethylated probe) were employed. Pair-wise comparisons between the combined PE status and NO_x_ exposure groups were performed using a contrast matrix (for a summary of the different comparisons made, see Supplemental Material, Figure S1). Empirical Bayes smoothing was applied to the standard errors with a robust selection against outlier sample variances. Further, p-values were adjusted for multiple comparisons for all CpGs by the Benjamini–Hochberg false discovery rate (FDR) method to obtain *q*-values. A *q*-value of 0.05 or lower was considered statistically significant. Analyses were performed including samples from: (1) both fetal sexes in the same analysis and (2) stratified for fetal sex for group comparisons that had significant DMPs.

Imprinted genes are expressed in a parent-of-origin-specific manner that was established in the parental germline and is caused by epigenetic processes involving DNA methylation and histone methylation. Many imprinted genes are involved in embryogenesis and placental development. All imprinted genes were removed from the results, and we used the following dataset to confirm imprinted genes (https://www.geneimprint.com/site/genes-by-species, accessed on 5 November 2021).

### 2.11. In Silico Analyses: Interaction Network Analyses and Gene Ontology Analyses for DNA Methylation

We used ConsensusPathDB (CPDB) (http://cpdb.molgen.mpg.de/, accessed on 5 November 2021) to search for potential gene regulatory interactions (induced network molecule analysis). In the analyses, we included genes that had CpGs with *q* < 0.1 in the DMP analyses. When comparing cases and controls with high NO_x_ exposure for female placentas, we had to use a cut-off value of *q* value < 0.05 due to the high number of DMPs.

Gene ontology (GO) analyses were run for genes that had CpGs with *q* < 0.1 in the DMP analyses. This was performed using the gometh (DMPs) function in the R package missMethyl [47]. A *q*-value of 0.05 or lower for the term in the GO analyses was considered statistically significant.

### 2.12. RNA Sequencing Analysis

Total RNA from placental biopsies, prepared as described above, was used to prepare libraries using the TruSeq^®^ Stranded mRNA Library Prep (20020594, Illumina, San Diego, CA, USA). Library preparation quality control (QC) was performed using LabChip DNA High Sensitivity Reagent kit (CLS138948, Perkin Elmer, Waltham, MA, USA) and DNA 1K/12K/Hi Sensitivity Assay LabChip (760517, Perkin Elmer). The library pool(s) were quantified using the QuantIT^®^ dsDNA HS Assay Kit (Q33120, Thermo Fischer, Waltham, MA, USA). The library pool was then sequenced as paired-end, 75-bp reads on a NovaSeq 6000 (Illumina).

Raw data were demultiplexed, and FASTQ files for each sample were generated using the bcl2fastq software (Illumina, San Diego, CA, USA). The FASTQ data were checked using the FastQC tool [48]. Alignment of reads were made to the reference genome sequence from the Ensembl database, the Human GRCh38. Assembly of the alignments into full transcripts and quantification of the expression levels of each gene and transcript were performed using the StringTie software [49]. There were 57,492 input genes, of which 1243 had zero expression, and 37,725 (65%) showed low expression and were removed using the filterByExpr function in DSEq2 [50]. Thus, 18,524 genes were included in the final analysis. Counts were normalized to Trimmed Mean of M-values (TMM) using limma. Differentially expressed genes (DEG) analysis was performed using Limma voom [46]. Since there were few individuals (N = 17), we only compared PE cases (N = 10) and controls (N = 7). Models were adjusted for fetal sex. A *q*-value of 0.05 or lower was considered statistically significant. Since no genes reached *q* < 0.1, we did not perform any GO analyses.

## 3. Results

### 3.1. Characteristics of the Study Participants 

First, we summarized the descriptive data of the participants and evaluated differences in characteristics between PE cases and controls using the χ2 test for categorical variables and a t-test for continuous variables (Table 1). There was a statistically significant lower mean gestational age among PE cases compared to controls (38.5 vs. 39.5 weeks, *p* = 0.014), and PE cases were also more likely to have had PE previously (*p* = 0.046).

### 3.2. Association between NO_x_ Exposure and PE Status

Associations between NO_x_ exposure during the first trimester (low or high exposure, low exposure is reference) and PE status were estimated with crude and adjusted logistic regression. The crude odds ratio (OR) for the association between exposure to ambient NO_x_ during the first trimester and PE was 2.3 (95% confidence interval (CI): 0.96, 5.6, *p* = 0.062). After controlling for maternal age, pregestational BMI, gestational age (linear), and fetal sex, the OR was 3.1 (95% CI: 1.1, 8.6), *p* = 0.028.

### 3.3. Association between Exposure to Ambient NO_x_ and PE Status with Gestational Age Acceleration/Deceleration

We investigated whether the combined PE status and NO_x_ exposure group was associated with GA acceleration/deceleration. By regressing the predicted GA to the chronological GA, the residual gives each placenta a value for GA acceleration/deceleration [41]. A positive value indicates that the placenta ages faster than expected, i.e., an accelerating placental epigenetic clock or GA acceleration, while a negative value indicates a GA deceleration.

Spearman correlations (R_s_) between predicted GA and chronological GA showed somewhat stronger correlations for PE cases (R_s_ = 0.64, *p* < 0.001) than for controls (R_s_ = 0.56, *p* < 0.001).

**Figure 2:**
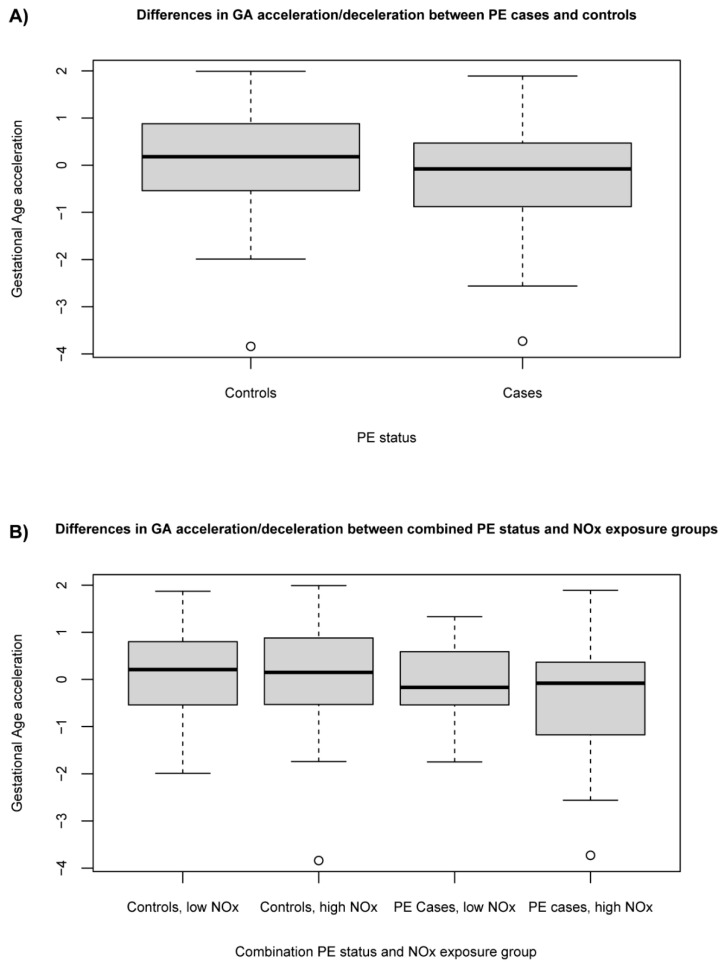
Gestational age (GA) acceleration/deceleration in (**A**) cases and controls and in (**B**) the different combinations of PE status and NO_x_ exposure group.

The PE cases demonstrated GA deceleration compared to controls, although this was not statistically significant (beta = −0.43, *p* = 0.057, controls are reference) (Figure 2A). Placentas from PE cases with high NO_x_ exposure showed significant GA deceleration compared to placentas from controls with low NO_x_ exposure (beta = −0.61, *p* = 0.035, model R^2^ = 0.03) (Figure 2B). However, the variable of combined PE status and NO_x_ group (as a categorical variable) was not statistically significant in the model (*p* = 0.20, model R^2^ = 0.04) (Supplemental Material, Table S1).

### 3.4. Differentially Methylated Positions Associated with PE Status and NO_x_ Exposure

We investigated whether the combined PE status and NO_x_ exposure group was associated with differential methylation of specific positions in the genome.

In the analyses including placentas of both fetal sexes, we identified six DMPs in the comparison between controls with low NO_x_ exposure vs. PE cases with high NO_x_ exposure, one DMP in the comparison between PE cases with low NO_x_ exposure vs. controls with high NO_x_ exposure and fourteen DMPs in the comparison between cases vs. controls, both with high NO_x_ exposure (*q* < 0.05, Table 2), making up a total of 19 CpGs showing differential methylation.

Two CpGs were statistically significant in both group comparisons: cg15999356 in the Yes1-associated transcriptional regulator gene (*YAP1*) and cg26672098 (located at chromosome 2p21, not annotated to any gene) showed lower methylation in the PE cases with high NO_x_ exposure group compared to the reference groups in both comparisons. All statistically significant CpG sites displayed lower methylation in the PE cases with high NO_x_ exposure group compared to the reference groups, except for one CpG site, which instead showed a higher methylation: cg02404739 in FAM111 trypsin-like peptidase B (*FAM111B*). To note is that two of the top DMPs, cg17283620 in 3-hydroxyanthranilate 3,4-dioxygenase (*HAAO*), and cg02171814 in serpin family F member 2 (*SERPINF2*), had nearby single-nucleotide polymorphisms (SNPs) with a frequency above 10% in the CEU reference population (Utah Residents with Northern and Western European Ancestry) [51,52]. These SNPs were rs12617051 (*HAAO*, minor allele frequency [MAF] 43%) and rs77305322 (*SERPINF2*, MAF 27%). *HAAO* rs12617051 was situated in the CpG site, while the *SERPINF2* rs77305322 was situated 35 base pairs away from the CpG site.

When stratifying for fetal sex, one DMP was seen for placentas with male fetuses in the comparison between PE cases with low NO_x_ exposure vs. controls with high NO_x_ exposure. This DMP was situated in the signal transducer and activator of transcription 3 (*STAT3*). For placentas with female fetuses, we identified 309 DMPs (*q* < 0.05) when comparing cases and controls with high NO_x_ exposure (Table 2). A total of 280 out of 309 (90%) statistically significant CpG sites (*q* < 0.05) displayed lower methylation in the PE cases with high NO_x_ exposure group compared to controls with low NO_x_ exposure. The top 50 statistically significant DMPs in the comparison between PE cases with low NO_x_ exposure vs. controls with high NO_x_ exposure for placentas with female fetuses are shown in Table 2 (top 13, based on *q*-values) and the Supplemental Material, Table S2 (ranked 14-50, based on *q*-values). Notably, cg09234983 in large tumor suppressor kinase 2 (*LATS2*) had a nearby SNP with a MAF above 10% in the CEU population. This SNP was rs9506597 and had a MAF of 28%. The distance between rs9506597 and the query base of the probe was 27 base pairs. There was also one DMP (cg16162930, not annotated to any gene) in the comparison between PE cases with low NO_x_ exposure vs. controls with high NO_x_ exposure for placentas with female fetuses. However, this CpG had a nearby SNP (rs2105225) with a MAF of 44% in the CEU population, situated at the CpG site.

### 3.5. In Silico Analyses: Interaction Network Analyses and Gene Ontology Analyses for DNA Methylation

The results from the analysis of gene regulatory interactions pointed to specific transcription factors (TFs). A central TF when comparing controls with low NO_x_ exposure vs. PE cases with high NO_x_ exposure was hepatocyte nuclear factor 4 alpha (*HNF4A*) (Supplemental Material, Figure S2A). When comparing cases and controls, both with high NO_x_ exposure, E2F transcription factor 4 (*E2F4*) and TATA-box binding protein associated factor 1 (*TAF1*) were central TFs (Supplemental Material, Figure S2B). Lastly, *TAF1* was a central TF when comparing low-exposed controls vs high-exposed PE in placentas from female fetuses (Supplemental Material, Figure S2C).

We performed GO analyses, including CpGs with *q* < 0.1 in the DMP analyses. The number of CpGs annotated to genes and thus included in the GO analyses were 123 in the comparison between controls with low NO_x_ exposure vs. PE cases with high NO_x_ exposure, and 264 in the comparison between cases and controls, both with high NO_x_ exposure. In the comparison between PE cases with low NO_x_ exposure vs. controls with high NO_x_ exposure, there was just one DMP with *q* < 0.1, and, thus, we did not perform any GO analysis. We found no significant hits in the GO analyses after the correction for multiple testing in any of these two group comparisons (FDR < 0.05) (Supplemental Material, Table S3).

For the sex-stratified analyses, for placentas from female fetuses, there were no DMPs with *q* < 0.1 in the DMP analyses comparing controls with high NO_x_ exposure vs. PE cases with high exposure, while the number of genes included in the GO analyses in the comparison between cases and controls with high NO_x_ exposure were 1465. For placentas from male fetuses, there was just one DMP with *q* < 0.1 in the comparison between PE cases with low NO_x_ exposure vs. controls with high NO_x_ exposure, and there were no DMPs with *q* < 0.1 in any of the other two group comparisons. Thus, no GO analyses were performed for placentas from male fetuses.

When stratifying for fetal sex, for placentas from female fetuses, one enriched pathway in the GO analyses, structural constituents of postsynapse, had an FDR < 0.1 when comparing cases and controls with high NO_x_ exposure (Supplemental Material, Table S3).

### 3.6. RNA Sequencing: Analysis of Differentially Expressed Genes and Gene Ontology

We investigated whether PE status was associated with differential gene expression.

Only samples with RIN > 7 were included in the RNA sequencing; thus, the sample size for the RNA-seq was only 17 individuals, and, due to the small sample size, no gender-stratified analyses were performed. Descriptive data for study participants included in RNA-seq are shown in Supplemental Material, Table S4. No genes reached statistical significance in the DEG analyses (*q* < 0.05). Top genes in the DEG analyses, ranked on unadjusted p-values, are shown in Table 3. None of the genes with DMPs (for both sexes together) showed a *p* < 0.05 in the DEG analyses.

## 4. Discussion

Our study has identified several differentially methylated placental genes that were associated with PE in relation to exposure to ambient NO_x_ during the first trimester. We also found sex differences, where placentas with female fetuses showed more differentially methylated placental genes than placentas with male fetuses. Placentas from PE cases with high NO_x_ exposure showed a significant gestational age deceleration compared to placentas from normotensive controls with low NO_x_ exposure, suggesting a state of placental villous immaturity [53].

The DNA methylation analysis indicated DNA hypomethylation in locations associated with several genes, of which many have been investigated in tumor growth and metastasis. Their specific role in placentation may remain unclear, but reactivation of embryonic developmental processes such as those involved in placental growth and invasion have been suggested to explain the molecular mechanisms in tumor growth and metastasis, depicting many similarities between the two phenomena [54,55,56]. Accordingly, it is interesting to find hypomethylation of two members of the SH3 domain protein family (*SH3D21* and *YAP1*) as well as *RPRD1B* in placentas from PE cases with high NO_x_ exposure compared to controls with low NO_x_ exposure. These three genes have been shown to play significant roles in regulation of cell proliferation, migration, and even differentiation in cancer. In trophoblasts, *YAP1* has been shown to regulate villous cytotrophoblasts proliferation and stemness, thus suppressing trophoblast fusion (to create syncytium) and even limiting extra villous trophoblast differentiation [57]. Inhibition or degradation of *YAP1*, on the other hand, can lead to trophoblast apoptosis [58] or decreased trophoblast invasion [59], indicating the significance of balance in YAP1 activity in different trophoblast cell types for effective placentation. Recent studies found significantly lower levels of YAP1 mRNA expression [60] and protein levels [61] in severe PE compared to uncomplicated pregnancies. Taken together, hypomethylation of *YAP1* may play a role in trophoblast fusion and proliferation in PE, thereby potentially also contributing to defective villous maturation and impaired placental function [62].

Another gene correlated with a hypomethylated position was *CACNA2D3*, which regulates voltage-dependent calcium channels. By introducing mitochondria-mediated apoptosis, *CACNA2D3* also plays a critical role in tumor suppression [63]. While calcium signaling and transfer in the placenta are complicated and significant for fetal development [64], altered expression of L-type voltage-dependent calcium channels in pregnancy disorders such as PE has been associated with depolarization in arterial smooth muscles and hypoxic vasoconstriction in the placenta [64,65]. Furthermore, intracellular alterations in calcium homeostasis have been shown in PE, where reports show reduced levels of the calcium-binding messenger calmodulin [66] and the downstream second messenger calcium/calmodulin-dependent kinase IV (*CAMK4*) [67]. Reduced levels of CAMK4 suppresses the proliferation and migratory capacity of trophoblasts, along with increased apoptosis [67]. Lastly, the hypomethylated DMP cg26672098 is not linked to any gene but located on chromosome 2p21. It was significant in the comparison between PE cases and controls, both with high NO_x_ exposure. This CpG has previously been found to be hypomethylated in association with early-onset PE [68].

When comparing PE cases and controls, both with high NO_x_ exposure, many of the genes were hypomethylated and previously associated with changes in actin cytoskeleton and cell proliferation, as well as migration and metastasis in cancer. For instance, studying *PEBP1* in cancer showed that it controls the cell cytoskeleton by interacting with actin, as well as regulating various signaling pathways and GTPases upstream of actin cytoskeleton regulation [69]. These changes in the cytoskeleton can be associated with membrane changes in processes from cell migration to vesicle formation and endocytosis [69]. In the placenta, PEBP1 has been suggested to regulate trophoblast migration and is expressed in villous cytotrophoblasts while its expression is translocated to the syncytium in PE placentas [70]. Due to syncytiotrophoblast stress in PE, there is an increased production and shedding of vesicles compared to normal pregnancies [71]. A key depolymerizer of actin filaments also found in a hypomethylated region in PE is *MICAL1*, which regulates actin cytoskeleton dynamics in a variety of processes from cytokinesis [72] to invasion and mobility in cancer [73]. Additionally, downregulation of *MICAL1* in PE placentas has been observed along with increased trophoblast proliferation [74]. In this comparison, we also saw *YAP1*, which was discussed earlier, and *MB21D2*, whose overexpression facilitates cell proliferation and invasion in cancer, while its suppression leads to apoptosis [75]. Another gene in a hypomethylated position is CD163, which is mostly known for being expressed on macrophages and endocytoses hemoglobin/haptoglobin complexes [76]. In the placenta, CD163 is expressed on the anti-inflammatory placental Hofbauer cells [77], and reduced numbers of these cells as well as down-regulation of *CD163* expression have previously been reported in PE [78,79]. This would result in a reduction of macrophage functions, such as promoting anti-inflammatory and pro-angiogenic responses in the placenta.

Our analysis also indicated three specific hypomethylated positions associated with *HAAO*, *SUPT3H,* and *SERPINF2*, which were not directly relevant to the above-mentioned mechanisms but still intriguing. For instance, in pregnancy, tryptophan catabolism is required to regulate the maternal immune reaction towards an implanting fetus [80]. In PE, tryptophan catabolism is deregulated, and *HAAO* mRNA levels are decreased [81,82], which may play some role in maternal–fetal tolerance. Tryptophan levels have also been shown to be decreased in PE, along with kynurenine pathway enzymes that break down tryptophan [82]. Tryptophan is also a precursor for the neurotransmitter serotonin, which is a critical player in fetal brain development [83]. Another gene, *SUPT3H*, considered to be important in the regulation of transcription [84], can, if mutated, cause placental defect and fetal mortality in mice [85]. The other gene with a unique role was *SERPINF2*, which is a plasmin inhibitor [86], but it also has a role in vascular remodeling [87]. Interestingly, it has been reported to be increased in maternal plasma in PE and co-localized with fibrin deposits in the PE placenta [88]. The last one, *PLCXD2,* is a phospholipase located in the nucleus with no known connection to the placenta or pregnancy, but a novel intronic SNP has recently been associated with risk for several cancers [89]. The only hypermethylated position in this comparison was associated with *FAM111B*, a nuclear serine protease that can promote proliferation, migration, and invasion in lung cancer, while its suppression induces cell apoptosis in cancer [90,91].

There were also a few DMPs in the comparison between PE cases with low NO_x_ exposure and controls with high NO_x_ exposure. Since this comparison looks at cases and controls with different exposure levels, there is no rationale or relevant scientific question for any further evaluation. However, the gene found was sodium channel and clathrin linker 1 (*SCLT1*), which is an adaptor protein that is involved in linking clathrin to sodium channels and therefore necessary for receptor endocytosis [92]. It has not previously been associated to PE or the placenta, but placenta-derived extracellular vesicles have been shown to be taken up by endothelial cells through clathrin-dependent endocytosis [93].

Overall, DNA methylation analysis of PE placentas in relation to exposure to high ambient NO_x_ indicate a potential deregulation in cell proliferation, differentiation, and migration associated with altered cytoskeletal dynamics and cell signaling. These changes may underlie the lower invasive capacity of the trophoblasts seen during implantation at Stage 1 of PE development, resulting in impaired placental perfusion [94]. Later, these changes can contribute to defective morphology and altered metabolism and immune response that are hallmarks of Stage 2 in PE [8]. In addition, our DNA methylation analysis indicated DNA hypomethylation, and this is in line with a recent systematic review demonstrating an association between exposure to ambient air pollution during pregnancy and global loss of methylation in the umbilical cord blood and placenta [95]. In addition, this report indicated that early pregnancy is the most vulnerable period when it comes to exposure.

For the female placentas comparing PE cases and controls, both with high NO_x_ exposure, our analysis mainly indicated hypomethylated positions. We identified several genes about which very little is known in the literature regarding their function and expression, and many of them had no known connection to pregnancy, the placenta, or PE. Information regarding expression in different tissues was instead available to us at the online databases The Human Protein Atlas (www.proteinatlas.org [96]) and the GeneCards (www.genecards.org, accessed on 5 November 2021 [97]). Several of the genes were connected to cell and/or vesicle migration and to the cytoskeleton. Again, making a comparison to tumor cells, the process of metastasis and tumor mobility has similarities to placental growth and invasion. One of the key elements in tumor mobility is the cytoskeleton that undergoes active change during this process. This results in changes in plasma membrane and in cellular balance between adhesion and mobility. Members of the tetraspanin family have been shown to play important roles in cytoskeleton dynamics during tumor metastasis and in the regulation of tumor microenvironment [98]. Interestingly, we found hypomethylation of *TSPAN4*, a member of the tetraspanin family, that has recently been shown to play a vital role in the formation of migrasomes, which are cellular organelles that form in migrating cells [99]. Additionally, *TSPAN4* has been shown to be expressed in placenta in different types of trophoblasts, as well as Hofbauer cells [96]. Hofbauer cells are of placental origin and have recently been shown to have microbicidal capacity as well as a possible role in placental angiogenesis and remodeling [100], which are functions that would require mobility. Two other hypomethylated genes, *ACTR3C* and *KRT24*, are both involved with the cytoskeleton. Protein ARP11, produced by ACTR3C, is an intracellular protein expressed in the placenta [96]. It is involved in regulation of the actin cytoskeleton [101] and in early formation of endosomes by being part of the dynein–dynactin motor complex [102]. Also involved with motor complexes is TRAK1, a mitochondrial adaptor protein that is essential for mitochondrial trafficking. When mitochondria move along microtubules, they are attached to TRAK proteins that link them to the two motor complexes kinesin-1 and dynein–dynactin driving the transport [103,104]. Another hypomethylated gene, *KRT24*, is highly expressed in trophoblast cells in the placenta [105] and forms the intermediate filaments that is part of the cytoskeleton. Yet another hypomethylated gene found to be expressed in the placenta is *RAB5C* [96]. It is an intracellular protein that is a member of the RAS oncogene family, located in endosomes and thought to be involved in docking and/or fusion of vesicles [106]. Not directly associated with the above-mentioned mechanisms, the peroxisomal membrane protein SLC25A17, known as a coenzyme A transporter and vital for functional peroxisomes [107], was also hypomethylated and belongs to mitochondrial solute carriers. It is found to have cytoplasmic and membrane expression in all tissues, including trophoblasts in placenta [96]. Finally, our analysis showed hypomethylation at a position associated to *LATS2*, which is a primary upstream negative regulator of *YAP1* [108], again pointing at the significance of balance in YAP1 activity for normal placenta development.

Hypermethylation was found at positions associated with genes *RTN4RL1* and *PRAC*. The first one, *RTN4RL1*, is a cell surface protein that is expressed at low levels in the placenta and predominantly in the brain [96]. Signaling via RTN4RL1 leads to activation of Rho and downstream restriction of actin polymerization, thereby regulating actin cytoskeleton during neural development to control the number of synapses formed [109]. The second one, *PRAC*, is a small nuclear protein and not reported to be expressed in the placenta. Instead, it is previously identified as prostate cancer susceptibility genes *PRAC1* and *PRAC2*, specifically over-expressed in human prostate and colon cancer [110].

For pregnancies with male fetuses, one DMP was detected in the comparison between PE cases with low NO_x_ exposure and controls with high NO_x_ exposure. Although we see no rationale or relevant scientific question for evaluating this group comparison, the gene *STAT3* is still of interest for PE. *STAT3* is a transcription factor and mediates expression of a variety of genes in response to cell stimuli, and thus plays a key role in many cellular processes such as cell growth and apoptosis. Decreased *STAT3* expression and activation have been suggested to play an important role in the pathogenesis of PE [111,112,113].

Our data further supports sexual dimorphism in the placenta and an association between PE and fetal sex. Female placentas are suggested to have a growth trajectory and invasion that is generally slower compared to male placentas [114,115], which is connected to cytoskeleton and migration. It is also suggested that disturbances, or in-utero insults, during early placenta development may affect growth of the female fetus more than the male, which is due to differences in placental growth [115]. Additionally, placental function during gestation is different between female and male placentas when it comes to strategies for metabolism and transport of nutrients, where male placentas have higher nutrient transfer capacity [114,116].

A gestational age deceleration among PE placentas exposed to high NO_x_ resembles reports about placental delayed villous maturation or villous immaturity associated with placental insufficiency, fetal hypoxia, term fetal death, and fetal growth restriction [117]. Normal maturation of the placenta involves formation of the new terminal villi and syncytium in response to increasing demands from the fetus. These are all activities that require changes in the cellular cytoskeleton. Villous immaturity is characterized by reduced formation of terminal villi [118], resulting in impaired placental function. In PE, there is evidence of altered syncytium homeostasis with reduced trophoblast proliferation, a reduced level of trophoblast fusion to create new syncytium, increased syncytiotrophoblast apoptosis, and reduced production of placental growth factor (PlGF) [119,120]. This is in accordance with our DNA methylation results, indicating that cytoskeletal dynamics as well as trophoblast function/apoptosis seem to be altered in PE cases exposed to high levels of NO_x_ and might be contributing to reduced maturation of the placenta. Placental age deceleration has previously been associated with maternal cardio-metabolic factors such as pre-pregnancy obesity and high blood pressure in women carrying male fetuses [121], further supporting sexual dimorphism. Interestingly, these are also known maternal risk factors for developing PE [122]. Additionally, exposure to a negative pregnancy environment associated with insulin-treated gestational diabetes or maternal Sjögren’s syndrome have been linked to epigenetic age deceleration in umbilical cord blood [123]. Early-onset, but not late-onset, PE has previously been associated with placental age acceleration [19,124]. However, in this study, very few PE cases belong to early-onset PE (1 out of 29 for all cases, and 1 out 19 for high NO_x_ exposure). Taken together, our data suggest a scenario where early-onset PE with poor placentation, hypoxia, and oxidative stress result in compensatory mechanisms such as accelerated villous maturation and epigenetic age acceleration, while late-onset PE, on the other hand, affected by maternal risk factors and air pollution exposure, results in placental immaturity and epigenetic age deceleration.

The CPDB analyses highlighted three specific transcription factors. The first one, *HNF4A*, is involved in several forms of cancer [125,126] and implicated in the formation of progenitor cells from stem cells. The next one, *E2F4*, is a transcriptional repressor involved in many different functions in the cell, such as cell cycle regulation and regenerative processes in stem cells and in cancer [127]. Both *HNF4A* and *E2F4* are implicated in signaling pathways related to tumorigenesis such as proliferation, invasion, self-renewal, and apoptosis [128]. The last one, *TAF1*, is an X-linked gene and the largest component of the basal transcription factor complex and therefore important for transcription in general. It has a binding site in the progesterone receptor gene promoter [129]. Variants of the gene or disrupted expression are implicated in neurodevelopment [130,131] but mutated variants of *TAF1* have also been implicated in cancer [132]. Additionally, here, we suggest that the impact these transcriptions factors have on molecular mechanisms in tumor growth and metastasis can be extrapolated to similar processes during placenta formation.

Several important limitations of this study need to be considered. Firstly, due to the low quality of RNA for most of the placenta samples, the power of the DEG analyses was very low, and it was not possible to expand our analysis of DNA methylation by correlating it to gene expression on a genome-wide scale. Secondly, although our findings of differential DNA methylation associated with ambient NO_x_ exposure during the first trimester and PE were assessed on mostly term placentas, it has been postulated and confirmed that DNA methylation levels increase during the first trimester to reach a consistent level during second and third trimesters [133,134]. Thirdly, the current investigation was limited by modeling only NO_x_ exposure and no other pollutants such as PM2.5 was considered due to practical constraints in modeling other ambient air pollutants during exposure assessment. Moreover, the modeled concentration of NO_x_ pertains to ambient air pollution at residence level but not accounting for total exposure, including indoor, occupational, and commuting exposures, which is a common and inherent limitation of epidemiologic studies of air pollution and PE [13]. Fourthly, we did not validate the findings in a validation cohort. However, it was not possible to find a relevant validation cohort consisting of placentas from PE cases and controls with data on NO_x_ exposure. Lastly, it was not possible to do sub-analysis by differentiating between early- and late-onset PE [8] due to a small sample size for the early-onset group. Additionally, four of the DMPs contained SNPs in and around the CpG site, which could bias the results due to impaired base extension since the probe cannot hybridize properly. This influence is stronger the closer the SNP is located to the CpG site. The SNPs in the *HAAO* cg17283620 and in the non-annotated cg16162930 were situated by the CpG sites, and this warrants caution. Thus, the results for these CpG sites are likely to be influenced by some genetic variation, but we estimate it to be like the CEU population. However, the two other SNPs (in *SERPINF2* and *LATS2*) did not include any SNPs nearby the CpG site.

The key strengths of this study are its access to a unique biobank designed to study PE and the individual exposure assessment by a local-scale high-resolution dispersion modeling. Additionally, since maternal smoking during pregnancy alters DNA methylation [135,136], we only included mothers who never smoked by their self-report. In addition, placenta is regarded as an appropriate tissue for DNA methylation analysis [137].

## 5. Conclusions

This study has enhanced the scientific understanding of how early pregnancy exposure to high concentrations of ambient NO_x_ affect placental DNA methylation in PE. These changes resulted in placental immaturity and showed that sexual dimorphism may be an important factor in the etiology.

## Figures and Tables

**Table 1:** Description of study participants ^a^ and test of differences in characteristics between preeclampsia (PE) cases and controls ^b^.

Characteristics	PE Cases, N = 29 (%)	Controls, N = 82 (%)	*p*-Value
Maternal age (years)	31.2 ± 5.6	30.2 ± 3.9	0.30
<35	20 (69.0)	70 (85.4)	0.053
35≤	9 (31.0)	12 (14.6)	
Maternal pregestational body mass index (BMI) (kg/m^2^)			
<18.5	0 (0)	1 (1.2)	0.062
18.5–24.9	11 (37.9)	44 (53.7)	
25–29.9	7 (24.1)	25 (30.5)	
30≤	11 (37.9)	12 (14.6)	
Maternal parity			
Nullipara	17 (58.6)	60 (73.2)	0.26
Primipara	10 (34.5)	16 (19.5)	
Multipara	2 (6.9)	6 (7.3)	
Previous gestational hypertension	0 (0)	2 (2.4)	0.40
Previous preeclampsia	6 (20.7)	6 (7.3)	0.046
Mode of delivery			
Vaginal	22 (75.9)	67 (81.7)	0.50
Caesarean	7 (24.1)	15 (18.3)	
Fetal sex			
Male	16 (55.2)	43 (52.4)	0.80
Female	13 (44.8)	39 (47.6)	
Preterm birth (<week 37)	4 (13.8)	4 (4.9)	0.11
Fetal weight (g)	3485 ± 562	3282 ± 753	
Year of birth			
2008	6 (20.7)	15 (18.3)	0.11
2009	8 (27.6)	21 (25.6)	
2010	2 (6.9)	0	
2011	2 (6.9)	19 (23.2)	
2014	5 (17.2)	13 (15.9)	
2015	6 (20.7)	14 (17.1)	
Gestational age by week	38.5 ± 2.1	39.5 ± 1.7	0.014

^a^ Mean and standard deviation (SD) are presented for continuous variables, and numbers (percentages) are presented for categorical variables. ^b^ Differences between PE cases and controls were analysed with an χ2 test for categorical variables and with a t-test for continuous variables.

**Table 2:** Differentially methylated positions (ranked by *q*-value) between comparisons of combinations of preeclampsia (PE) status and ambient nitrogen oxides (NO_x_) exposure group.

CpG	Chr	Gene	Gene Name	2^log FC^ (95% CI) ^a^	Beta ^b^	*q*-Value
**Controls with low NO_x_ exposure (reference) vs. PE cases with high NO_x_ exposure**						
cg27596779	5	*NA* ^c^		0.78 (0.72, 0.86)	0.64	0.028
cg26672098	2	*NA*		0.75 (0.68, 0.83)	0.34	0.028
cg07806361	3	*CACNA2D3*	*Calcium voltage-gated channel auxiliary subunit alpha 2 delta 3*	0.62 (0.53, 0.73)	0.72	0.031
cg23935220	20	*RPRD1B*	*Regulation of nuclear pre-mRNA domain containing 1B*	0.77 (0.70, 0.85)	0.18	0.034
cg05694331	1	*SH3D21*	*SH3 domain-containing protein 21*	0.78 (0.71, 0.85)	0.68	0.034
cg15999356	11	*YAP1*	*Yes1-associated transcriptional regulator*	0.77 (0.70, 0,85)	0.66	0.036
**Controls with high NO_x_ exposure (reference) vs. PE cases with high NO_x_ exposure**						
cg17283620	2	*HAAO*	*3-hydroxyanthranilate 3,4-dioxygenase*	0.1 (0.05, 0.21)	0.14	0.005
cg26672098	2	*NA*		0.74 (0.67, 0.81)	0.34	0.006
cg24832457	15	*NA*		0.73 (0.66, 0.81)	0.56	0.010
cg15534461	12	*PEBP1*	*phosphatidylethanolamine binding protein 1*	0.70 (0.62, 0.8)	0.69	0.021
cg18531351	15	*NA*		0.77 (0.7, 0.84)	0.61	0.021
cg02404739	11	*FAM111B*	*FAM111 trypsin-like peptidase B*	2.58 (1.85, 3.63)	0.16	0.021
cg07715379	3	*PLCXD2*	*phosphatidylinositol specific phospholipase C X domain containing 2*	0.76 (0.68, 0.84)	0.5	0.042
cg18235274	12	*CD163*	*CD163 molecule, also known as M130*	0.68 (0.59, 0.78)	0.83	0.042
cg23502295	6	*MICAL1*	*microtubule associated monooxygenase, calponin, and LIM domain containing 1*	0.77 (0.70, 0.85)	0.83	0.042
cg15999356	11	*YAP1*	*Yes1 associated transcriptional regulator*	0.77 (0.74, 0.87)	0.66	0.042
cg07249517	3	*MB21D2*	*Mab-21 domain containing 2 also known as:C3orf59*	0.80 (0.74, 0.87)	0.56	0.047
cg08493590	6	*SUPT3H*	*SPT3 homolog, SAGA, and STAGA complex component*	0.74 (0.66, 0.83)	0.66	0.047
cg02171814	17	*SERPINF2*	*serpin family F member 2*	0.74 (0.66, 0.82)	0.61	0.047
**Controls with high NO_x_ exposure (reference) vs. PE cases with low NO_x_ exposure**						
cg18904784	4	*SCLT1*	*sodium channel and clathrin linker 1*	0.54 (0.45, 0.67)	0.02	0.031
**Female pregnancies only—Controls with high NO_x_ exposure (reference) vs. PE cases with high NO_x_ exposure**						
cg11398400	17	*RTN4RL1*	*reticulon 4 receptor like 1*	2.71 (2, 3.66)	0.95	0.011
cg09234983	13	*LATS2*	*large tumor suppressor kinase 2*	0.59 (0.5, 0.69)	0.39	0.011
cg22343476	1	*NA*		0.46 (0.36, 0.59)	0.24	0.011
cg08568550	11	*C11orf16*	*chromosome 11 open reading frame 16*	0.65 (0.56, 0.74)	0.57	0.011
cg24474409	7	*ACTR3C*	*actin related protein 3C*	0.47 (0.37, 0.59)	0.74	0.011
cg17764946	3	*TRAK1*	*trafficking kinesin protein 1*	0.46 (0.36, 0.59)	0.88	0.011
cg04219544	17	*KRT24*	*keratin 24*	0.57 (0.48, 0.68)	0.3	0.011
cg24551459	17	*RAB5C*	*member RAS oncogene family*	0.46 (0.35, 0.59)	0.74	0.011
cg14620234	9	*NA*		0.67 (0.59, 0.76)	0.31	0.015
cg11173246	11	*TSPAN4*	*tetraspanin 4*	0.44 (0.33, 0.57)	0.3	0.015
cg08157194	22	*SLC25A17*	*Solute Carrier Family 25 Member 17*	0.6 (0.51, 0.71)	0.55	0.016
cg04574034	17	*PRAC*	*PRAC small nuclear protein*	2.88 (2.03, 4.08)	0.8	0.016
cg24874090	8	*NA*		0.62 (0.53, 0.73)	0.64	0.019
**Female pregnancies only—Controls with high NO_x_ exposure (reference) vs. PE cases with low NO_x_ exposure**						
cg16162930	1	*NA*		0.10 (0.05, 0.20)	0.57	0.015
**Male pregnancies only—Controls with high NO_x_ exposure (reference) vs. PE cases with low NO_x_ exposure**						
cg09804439	17	*STAT3*	Signal transducer and activator of transcription 3	0.15 (0.09, 0.26)	0.02	0.004

Abbreviations: Chr, chromosome; CI, confidence interval; *q*-value, false discovery rate (FDR)—adjusted *p*-value using the Benjamini–Hochberg method; FC, fold change. ^a^ 2^logFC^, binary logarithmic fold change. LogFC denotes β_1_ from the following robust regression model: M-value = β_1_ × PE/NO_x_ group + β_2_ × DNA concentration + β_3_ × pregestational BMI + β_4_ × gestational age + β_5_ × fetal sex + β_6_ × estimated fraction Hofbauer cells + β_7_ × estimated fraction Syncytiotrophoblast cells. For female placentas, the models look as follows: M-value = β_1_ × PE/NO_x_ group + β_2_ × DNA concentration + β_3_ × gestational age + β_4_ × estimated fraction Hofbauer cells + β_5_ × estimated fraction Syncytiotrophoblast cells. ^b^ Average methylation state, expressed as Beta-value, for all study participants, ranging from 0 to 1 (1 means fully methylated). ^c^ NA, not annotated, i.e., the CpG is not present in any known gene.

**Table 3:** Differentially expressed genes (ranked by *q*-value) between comparisons of combinations of PE status and NO_x_ group.

Gene	Gene Name	2^logFC^ (95% CI) ^a^	*p*-Value (Unadjusted)	*q*-Value
*ESCO2*	establishment of sister chromatid cohesion N-acetyltransferase 2	2.4 (1.7, 3.3)	0.00002	0.33
*MUC20P1*	mucin 20, cell surface associated pseudogene 1	19.0 (6.1, 59.4)	0.00003	0.33
*LINC01003*	long intergenic non-protein coding RNA 1003	0.58 (0.47, 0.73)	0.0001	0.64
*KCNA4*	potassium voltage-gated channel subfamily A member 4	3.6 (1.9, 6.7)	0.0004	0.94
*TTC3*	tetratricopeptide repeat domain 3	1.4 (1.2, 1.6)	0.0005	0.94
*ABCC13*	ATP binding cassette subfamily C member 13 (pseudogene)	0.36 (0.21, 0.60)	0.0006	0.94
*GNAI1*	G protein subunit alpha i1	0.72 (0.61, 0.86)	0.0009	0.94
*SRRM3*	serine/arginine repetitive matrix 3	0.44 (0.27, 0.69)	0.0013	0.94
*AL451007.2*		0.29 (0.15, 0.58)	0.0014	0.94
*PCDHB18P*	protocadherin beta 18 pseudogene	3.3 (1.7, 6.7)	0.0016	0.94

^a^ 2^logFC^, binary logarithmic fold change. LogFC denotes β_1_ from the following robust regression model: TMM = β_1_ × PE status + β_2_ × Sex.

## Data Availability

The data underlying this article cannot be shared publicly due to the privacy of individuals that participated in the study. The datasets related to the clinical parameters and the DNA analysis are available upon request to the corresponding author. However, the datasets related to individual exposure levels of air pollution are not readily available because of limitations set in the ethical permission stating that only the researchers involved in the project are allowed to access data. Requests to access the dataset should be directed to data-holders, Statistic Sweden, and The Swedish National Board of Health and Welfare for birth registry data.

## Supplementary Materials

The following are available online at https://www.mdpi.com/article/10.3390/toxics9120338/s1, Figure S1: Summary of groups based on a combination of pregnancy status (PE or normotensive control) and exposure to ambient NO_x_ (low or high NO_x_ exposure based on median split) (Table A) and the pair-wise comparisons performed in the DMP analyses, where comparisons that had DMPs (*q* < 0.05) are marked in bold (Table B), Figure S2: Potential gene regulatory interactions (induced network molecule analysis) including genes with a *q* < 0.1 in the DMP analyses. Gene interaction networks are shown in purple, while protein interaction networks are shown in yellow. Figure A shows a comparison of controls with low NO_x_ exposure vs. PE cases with high NO_x_ exposure, figure B shows a comparison of cases and controls, both with high NO_x_ exposure, and figure C shows a comparison of controls with low NO_x_ exposure vs. PE cases with high NO_x_ exposure in placentas from female fetuses. Table S1: Differences in gestational age between groups based on combinations of PE status and NO_x_ exposure, analysed using linear regression, Table S2: Differentially methylated positions (DMPs) when comparing controls with high NO_x_ exposure, (reference) vs. PE cases with high NO_x_ exposure in pregnancies with female fetuses. The twelve DMPs with lowest *q*-value are already presented in Table 2 in the main text, thus the DMPs presented here are the DMPs ordered 14–50 ranked by *q*-value. Table S3: Top Gene Ontology (GO) terms for analyses including genes with CpGs with *q* < 0.1 in the DMP analyses for different combined PE status and NO_x_ group comparisons, Table S4: Comparisons between the study populations included in the placental DNA methylation analyses and RNA sequencing analyses.
