# Supplementary material for: Early Pregnancy Exposure to Ambient Air Pollution among Late-Onset Preeclamptic Cases Is Associated with Placental DNA Hypomethylation of Specific Genes and Slower Placental Maturation
Source: Toxics. 2021 Dec 6;9(12):338. doi: 10.3390/toxics9120338 (PMC8708250; doi:10.3390/toxics9120338)
Supplement: Supplementary file 1 [file toxics-09-00338-s001.zip › toxics-1484218-supplementary.pdf]

Supplementary Materials: Early Pregnancy Exposure to Ambient Air Pollution among Late-Onset Preeclamptic Cases is Associated with Placental DNA Hypomethylation of Specific Genes and Slower Placental Maturation

Karin Engström, Yumjirmaa Mandakh, Lana Garmire, Zahra Masoumi, Christina Isaxon, Ebba Malmqvist, Lena Erlandsson and Stefan R. Hansson

Table S1. Differences in gestational age between combined PE status and NO_x exposure groups, analysed using linear regression.

PE status and NO_x group	beta[a]	Std error	*P*-value
1. Controls, low NO_x	reference	reference	reference
2. Controls, high NO_x	−0.14	0.23	0.53
3. Cases, low NO_x	−0.28	0.36	0.44
4. Cases, high NO_x	−0.61	0.28	0.035*
Model p-value			0.20

[a]Beta is derived from the following linear model: Epigenetic age = intercept + β_1* PE status and NO_x group combination (categorical).

Table S2. Differentially methylated positions (DMPs) when comparing controls with high NO_x exposure, (reference) vs. PE cases withd high NO_x exposure in pregnancies with female fetuses. The twelve DMPs with lowest q-value are already presented in Table 2 in the main text, thus the DMPs presented here are the DMPs ordered 14–50 ranked by q-value.

Cpg	Chr	Gene	Gene name	2^{logFC} (95% CI)[a]	Beta[b]	*p*-value	*q*-value
cg15812969	21	*USP25*	*ubiquitin specific peptidase 25*	0.71 (0.63, 0.80)	0.41	0.000000339	0.019
cg22356173	17	*CLUH*	*clustered mitochondria homolog*	0.59 (0.49, 0.71)	0.05	0.000000350	0.019
cg10926412	13	*NA*[c]		0.61 (0.51, 0.72)	0.40	0.000000385	0.020
cg25030347	17	*RPTOR*	*regulatory associated protein of MTOR complex 1*	0.43 (0.32, 0.57)	0.88	0.000000448	0.022
cg06343806	7	*NA*		0.62 (0.52, 0.73)	0.36	0.000000541	0.022
cg17936567	22	*TAB1*	*TGF-beta activated kinase 1 (MAP3K7) binding protein 1*	0.74 (0.67, 0.82)	0.28	0.000000549	0.022

cg13923903	22	*ZNRF3*	*zinc and ring finger 3*	0.70 (0.61, 0.79)	0.40	0.000000587	0.022
cg25141112	8	*LINC01298*	*long intergenic non-protein coding RNA 1298(LINC01298)*	0.71 (0.64, 0.80)	0.61	0.000000615	0.022
cg26187194	10	*NA*		2.91 (2.00, 4.23)	0.70	0.000000623	0.022
cg10590292	12	*BIN2*	*bridging integrator 2*	0.44 (0.33, 0.59)	0.68	0.000000626	0.022
cg23745445	15	*ANP32A*	*acidic nuclear phosphoprotein 32 family member A*	0.65 (0.56, 0.76)	0.23	0.000000628	0.022
cg18686217	3	*DAG1*	*dystroglycan 1*	0.67 (0.58, 0.77)	0.61	0.000000829	0.024
cg18014788	18	*PTPRM*	*protein tyrosine phosphatase, receptor type M*	0.52 (0.41, 0.65)	0.84	0.000000869	0.024
cg01234517	7	*ACTR3C*	*ARP3 actin-related protein 3 homolog C*	0.48 (0.37, 0.63)	0.79	0.000000930	0.024
cg08304968	20	*NA*		0.66 (0.56, 0.76)	0.53	0.000000945	0.024
cg04728805	5	*FAM53C*	*family with sequence similarity 53 member C*	0.64 (0.55, 0.75)	0.10	0.000000976	0.024
cg16849481	11	*SPDYC*	*speedy/RINGO cell cycle regulator family member C*	0.69 (0.61, 0.79)	0.39	0.000000995	0.024
cg06154002	14	*TDRD9*	*tudor domain containing 9*	2.47 (1.79, 3.41)	0.33	0.000001008	0.024
cg16414660	2	*DGKD*	*diacylglycerol kinase delta(DGKD)*	0.59 (0.49, 0.72)	0.70	0.000001046	0.024
cg01584478	4	*LRBA*	*LPS responsive beige-like anchor protein*	0.62 (0.52, 0.73)	0.43	0.000001080	0.024
cg11548591	9	*DAB2IP*	*DAB2 interacting protein*	0.54 (0.43, 0.67)	0.67	0.000001112	0.024
cg23502295	6	*MICAL1*	*microtubule associated monooxygenase, calponin and LIM domain containing 1*	0.68 (0.59, 0.78)	0.81	0.000001116	0.024
cg16013484	5	*NA*		0.59 (0.48, 0.71)	0.72	0.000001164	0.024
cg17971961	1	*KIAA0319L*	*KIAA0319 like*	0.67 (0.59, 0.78)	0.69	0.000001169	0.024
cg07249517	3	*MB21D2*	*Mab-21 domain containing 2*	0.70 (0.62, 0.80)	0.55	0.000001188	0.024
cg02712011	3			0.65 (0.56, 0.76)	0.11	0.000001233	0.024
cg25473467	6	*ANKRD6*	*ankyrin repeat domain 6*	0.62 (0.53, 0.74)	0.12	0.000001254	0.024
cg25569590	7	*COBL*	*cordon-bleu WH2 repeat protein*	0.55 (0.44, 0.68)	0.24	0.000001265	0.024
cg19581707	22	*SLC25A18*	*solute carrier family 25 member 18*	0.68 (0.59, 0.78)	0.30	0.000001281	0.024

cg22490758	11	*PC*	*pyruvate carboxylase*	0.71 (0.63, 0.81)	0.71	0.000001302	0.024
cg07326313	15	*NA*		0.56 (0.45, 0.69)	0.30	0.000001316	0.024
cg24025567	7	*CUX1*	*cut like homeobox 1*	0.68 (0.60, 0.79)	0.56	0.000001344	0.024
cg11071407	6	*PPARD*	*peroxisome proliferator activated receptor delta*	0.61 (0.50, 0.73)	0.61	0.000001397	0.024
cg05844827	17	*MSI2*	*musashi RNA binding protein 2*	0.62 (0.53, 0.74)	0.45	0.000001432	0.024
cg00821201	20	*DSTN*	*destrin, actin depolymerizing factor*	0.63 (0.53, 0.75)	0.51	0.000001509	0.024
cg12906748	11	*C11orf16*	*chromosome 11 open reading frame 16*	0.63 (0.54, 0.75)	0.56	0.000001512	0.024
cg25688280	6	*RANBP9*	*RAN binding protein 9*	0.69 (0.60, 0.79)	0.39	0.000001514	0.024

Abbreviations: Chr, chromosome; CI, confidence interval; *q*-value, False discovery rate (FDR)-adjusted *p*-value using the Benjamini-Hochberg method; FC, fold change. [a]2^{logFC}, binary logarithmic fold change. LogFC denotes β_1 from the following robust regression model: M-value= β_1 × PE/NO_x group + β_2 × DNA concentration + β_3 × gestational age + β_4 × estimated fraction Hofbauer cells + β_5 × estimated fraction Syncytiotrophobast cells.[b]Average methylation state, expressed as Beta-value, for all study participants, ranging from 0 to 1 (1 means fully methylated). [c] NA, not annotated, i.e., the CpG is not present in any known gene.

Table S3. Top Gene Ontology (GO) terms for analyses including genes with CpGs with *q*<0.1 in the DMP analyses for different combined PE status and NO_x group comparisons.

GO ID	Term	Total Nr of genes in Term	Nr of genes from DMPs with *q*<0.1	unadjusted *p*-value	*q*-value
Controls with low NO_x exposure(reference) vs. PE cases and high NO_x exposure					
GO:0072080	nephron tubule development	88	5	0.000027	0.30
GO:0061326	renal tubule development	90	5	0.000031	0.30
GO:0050508	glucuronosyl-N-acetylglucosaminyl-proteoglycan 4-alpha-N-acetylglucosaminyltransferase activity	3	2	0.000049	0.30
GO:0072009	nephron epithelium development	104	5	0.000053	0.30
GO:0009595	detection of biotic stimulus	29	3	0.000081	0.37
GO:0072006	nephron development	137	5	0.000187	0.56
GO:0072088	nephron epithelium morphogenesis	72	4	0.000198	0.56
GO:0072073	kidney epithelium development	135	5	0.000203	0.56
GO:0072028	nephron morphogenesis	74	4	0.000222	0.56
GO:0032490	detection of molecule of bacterial origin	11	2	0.000371	0.71
GO:0072257	metanephric nephron tubule epithelial cell differentiation	7	2	0.000402	0.71
GO:0072307	regulation of metanephric nephron tubule epithelial cell differentiation	7	2	0.000402	0.71
Controls with high NO_x exposure (reference) vs. PE cases with high NO_x exposure					
GO:0032570	response to progesterone	44	4	0.000270	1
GO:0051016	barbed-end actin filament capping	20	3	0.000544	1
GO:0005996	monosaccharide metabolic process	270	8	0.000620	1
GO:2000813	negative regulation of barbed-end actin filament capping	4	2	0.000668	1
GO:0009595	detection of biotic stimulus	29	3	0.000729	1
GO:2000812	regulation of barbed-end actin filament capping	5	2	0.000790	1
GO:0001820	serotonin secretion	9	2	0.000875	1
GO:0016861	intramolecular oxidoreductase activity, interconverting aldoses and ketoses	8	2	0.000988	1
GO:0032490	detection of molecule of bacterial origin	11	2	0.001350	1
GO:0019318	hexose metabolic process	230	7	0.001428	1
Female pregnancies only - Controls with high NO_x exposure (reference) vs. PE cases with high NO_x exposure					
GO:0099186	structural constituent of postsynapse	10	6	0.000003	0.06
GO:0098918	structural constituent of synapse	16	7	0.000013	0.15
GO:0008089	anterograde axonal transport	48	9	0.000106	0.66
GO:0036464	cytoplasmic ribonucleoprotein granule	209	19.5	0.000117	0.66
GO:0035770	ribonucleoprotein granule	220	19.5	0.000218	0.97
GO:0030705	cytoskeleton-dependent intracellular transport	178	18	0.000274	0.97
GO:0035033	histone deacetylase regulator activity	4	3	0.000332	0.97
GO:0003727	single-stranded RNA binding	93	11	0.000343	0.97
GO:0007006	mitochondrial membrane organization	130	13	0.000485	1
GO:1904115	axon cytoplasm	57	9	0.000503	1

Table S4. Comparisons between the study populations included in the placental DNA methylation analyses and RNA sequencing analyses [a].

Characteristics	DNA methylation (n = 111)	RNA sequencing (n = 17)
Preeclampsia	29 (26.1)	10 (58.9)
Maternal age		
<35	90 (81.1)	14 (82.4)
35≤	21 (18.9)	3 (17.6)
Maternal pregestational BMI		
<18.5	1 (0.9)	0
18.5-24.9	55 (49.5)	7 (41.2)
25-29.9	32 (28.8)	2 (11.8)
30≤	23 (20.7)	8 (47.0)
Maternal Parity		
Nullipara	77 (69.4)	10 (58.8)
Primipara	26 (23.4)	4 (23.5)
Multipara	8 (7.2)	3 (17.6)
Previous Gestational Hypertension	2 (1.8)	0
Mode of delivery		
Vaginal	89 (80.2)	16 (92.3)
Cesarean	22 (19.8)	1 (5.9)
Fetal sex		
Male	59 (53.2)	8 (47.0)
Female	52 (46.8)	9 (53.0)
Gestational age by days	274.5±12.8	277 ± 7.8
Preterm birth	8 (7.2)	0
Fetal weight (g)	3432±621	3655±383
Year of birth		
2008	21 (18.9)	5 (29.4)
2009	29 (26.1)	4 (23.5)
2010	2 (1.8)	1 (5.9)
2011	21 (18.9)	4 (29.4)
2014	18 (16.2)	1 (5.9)
2015	20 (18.0)	2 (11.8)

[a] Mean, SD are presented for continuous variables, and numbers (percentages) are presented for categorical variables.

A)

⬇ PE status NO_x exposure ➡	Low	High
Control	Group 1 N = 45	Group 2 N = 37
Case	**Group 3 N = 10**	**Group 4 N = 19**

B)

Pair-wise group comparisons		
Controls, high NO_x	VS.	Controls, low No_x
PE cases, low NO_x	VS.	Controls, low No_x
**PE cases, high NO_x**	**VS.**	**Controls, low No_x**
**PE cases, low NO_x**	**VS.**	**Controls, high No_x**
**PE cases, high NO_x**	**VS.**	**Controls, high No_x**
PE cases, high NO_x	VS.	PE cases, low NO_x
PE cases, low NO_x	VS.	Controls, low No_x

Figure S1. Summary of groups based on a combination of pregnancy status (PE or normotensive control) and exposure to ambient NO_x (low or high NO_x exposure based on median split) (Table A) and the pair-wise comparisons performed in the DMP analyses, where comparisons that had DMPs (q<0.05) are marked in bold (Table B).

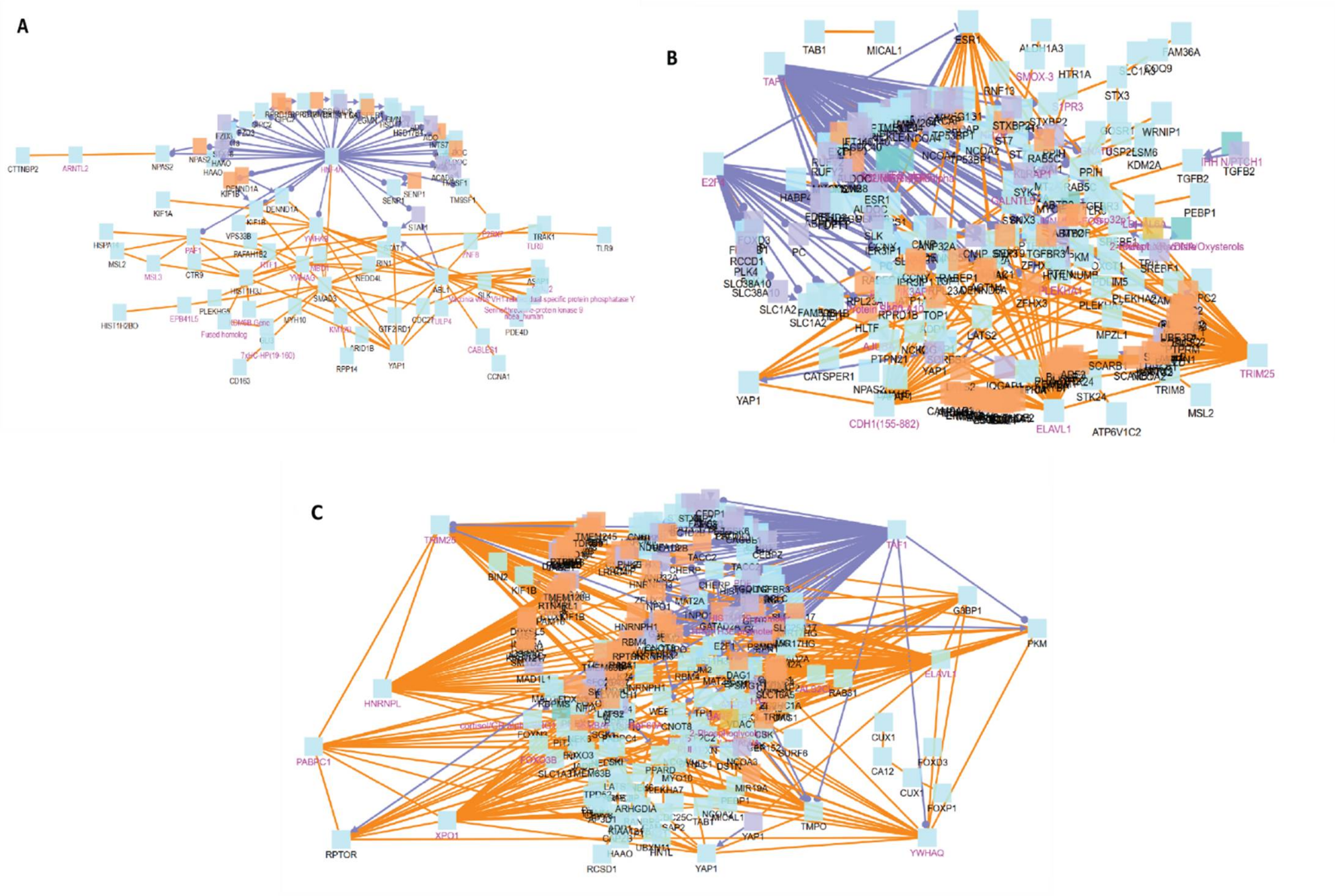

Figure S2. Potential gene regulatory interactions (induced network molecule analysis) including genes with a *q*<0.1 in the DMP analyses. Gene interaction networks are shown in purple, while protein interaction networks are shown in yellow. Figure A shows a comparison of controls with low NO_x exposure vs. PE cases with high NOx exposure, figure B shows a comparison of cases and controls, both with high NO_x exposure, and figure C shows a comparison of controls with low NO_x exposure vs. PE cases with high NOx exposure in placentas from female fetuses.
